# Unravelling the Dermatological Potential of the Brown Seaweed *Carpomitra costata*

**DOI:** 10.3390/md19030135

**Published:** 2021-02-28

**Authors:** Patrícia Susano, Joana Silva, Celso Alves, Alice Martins, Helena Gaspar, Susete Pinteus, Teresa Mouga, Márcia Ines Goettert, Željko Petrovski, Luís B. Branco, Rui Pedrosa

**Affiliations:** 1 MARE—Marine and Environmental Sciences Centre, Polytechnic of Leiria, 2520-630 Peniche, Portugal; patricia_susano94@hotmail.com (P.S.); joana.m.silva@ipleiria.pt (J.S.); alice.martins@ipleiria.pt (A.M.); hmgaspar@fc.ul.pt (H.G.); susete.pinteus@ipleiria.pt (S.P.); 2 BioISI-Biosystems and Integrative Sciences Institute, Faculty of Sciences, University of Lisbon, 1749-016 Lisboa, Portugal; 3 MARE—Marine and Environmental Sciences Centre, ESTM, Polytechnic of Leiria, 2520-614 Peniche, Portugal; mougat@ipleiria.pt; 4 Cell Culture Laboratory, Postgraduate Programme in Biotechnology, University of Vale do Taquari-Univates, Lajeado, RS 95914-014, Brazil; marcia.goettert@univates.br; 5 LAQV-REQUIMTE, Departamento de Química, Faculdade de Ciências e Tecnologia da Universidade Nova de Lisboa, 2829-516 Caparica, Portugal; z.petrovski@fct.unl.pt (Ž.P.); l.branco@fct.unl.pt (L.B.B.)

**Keywords:** algae, anti-enzymatic, anti-inflammatory, antimicrobial, antioxidant, marine natural products, phenolic compounds, photoprotective, skincare

## Abstract

The ever-increasing interest in keeping a young appearance and healthy skin has leveraged the skincare industry. This, coupled together with the increased concern regarding the safety of synthetic products, has boosted the demand for new and safer natural ingredients. Accordingly, the aim of this study was to evaluate the dermatological potential of the brown seaweed *Carpomitra costata.* The antioxidant, anti-enzymatic, antimicrobial, photoprotective and anti-inflammatory properties of five *C. costata* fractions (F1–F5) were evaluated. The ethyl acetate fraction (F3) demonstrated the most promising results, with the best ability to scavenge 2,2-diphenyl-1-picrylhydrazyl (DPPH) radicals (EC_50_ of 140.1 µg/mL) and the capacity to reduce reactive oxygen species (ROS) production promoted by UVA and UVB radiation in 3T3 cells, revealing its antioxidant and photoprotective potential. This fraction also exhibited the highest anti-enzymatic capacity, inhibiting the activities of collagenase, elastase and tyrosinase (IC_50_ of 7.2, 4.8 and 85.9 µg/mL, respectively). Moreover, F3 showed anti-inflammatory potential, reducing TNF-α and IL-6 release induced by LPS treatment in RAW 264.7 cells. These bioactivities may be related to the presence of phenolic compounds, such as phlorotannins, as demonstrated by NMR analysis. The results highlight the potential of *C. costata* as a source of bioactive ingredients for further dermatological applications.

## 1. Introduction

Due to the growing awareness of the skin’s importance, and the interest in a young and healthy appearance, the skincare industry is one of the fastest growing markets in recent years. One of the main goals of this industry is the search for new natural ingredients with a wide range of bioactivities, e.g., anti-aging, antioxidant, anti-inflammatory, antimicrobial, photoprotective, wound healing, moisturizing and whitening properties, in order to produce new products to respond to the growing demand for safer naturally-derived formulations [1].

Skin aging is a slow and complex process, characterized by the appearance of wrinkles, pallor, loss of elasticity, dryness, sagging and pigmentation [2,3]. This biological process can occur either due to endogenous factors, which are associated with metabolism, hormones and genetic predisposition, or due to exogenous factors, which are often related with the exposure to chemicals, toxins, pathogens, smoking, poor diet and ultraviolet radiation (UVR) [2,4]. UVR is classified according to its emission wavelength as UVA (320–400 nm), UVB (290–320 nm) or UVC (200–290 nm). UVC are blocked by the ozone layer and, therefore, does not reach the Earth’s surface, whereas UVA and UVB can pass through the ozone layer, imposing damage to the skin [5].

Exposure to UVR originates photo-oxidation reactions, impairing the skin’s antioxidant defence mechanisms and consequent acceleration of photoaging events [6,7]. When these mechanisms are overloaded, it results in the accumulation of reactive oxygen species (ROS) triggering several dermatological disorders and, subsequently, aging. With an increase in ROS levels, mitogen-activated protein kinase (MAPKs) family proteins are activated, inducing the overexpression of activator protein 1 (AP-1), activating transcription factor 2 (ATF-2) and the nuclear transcription factor kappa (NF-κB).

AP-1 induces the expression of the matrix metalloproteinases (MMPs), collagenase (MMP1) and elastase (MMP12), responsible for the degradation of extracellular matrix molecules (ECM) such as collagen and elastin, respectively [8]. On the other hand, ATF-2 and NF-κB stimulate the transcription of interleukins (IL-1, IL-6, IL-8) and the tumour necrosis factor alpha (TNF-α) pro-inflammatory mediators responsible for several skin-related conditions, such as inflammation, melanoma, or cancer [9,10]. In addition to collagen and elastin, hyaluronic acid (HA) is also a very important ECM, being responsible for skin hydration maintenance and flexibility, while also being involved in the skin repair metabolism against UVR damage [11,12]. However, HA levels tend to decrease naturally over time due to the action of the enzyme hyaluronidase. Therefore, one of the main approaches to minimize skin aging is to increase the levels of HA, collagen, and elastin, while simultaneously decreasing the expression of MMPs [13].

Another contributing factor for skin aging is the excessive production of melanin. After exposure to UVR, melanin is produced in skin melanocytes as a physiological response to both environmental and hormonal factors [14]. Melanin is produced through the hydroxylation of l-tyrosine in l-DOPA (3,4-dihydroxy-l-phenylalanine) and subsequent oxidation of l-DOPA in dopaquinone through the enzyme tyrosinase [15]. However, the production of excessive melanin can lead to hyperpigmentation disorders, such as melasma, freckles and blemishes [16]. Thus, since tyrosinase plays a crucial role in the synthesis of melanin, its inhibition is considered an effective approach for the treatment of the hyperpigmentation and whitening of the skin [17,18].

In the production of new skincare formulations, it is also important to include bioactive compounds for the treatment of various skin disorders. The human skin microbiota is composed by several microorganisms [19], such as the bacteria *Staphylococcus epidermidis*, *Cutibacterium acnes* and the fungus *Malassezia furfur.* This microbiota is responsible for maintaining a healthy skin barrier through a symbiosis between skin-related microorganisms and the skin. However, when exposed to exogenous and/or endogenous factors, this microbiota may experience some changes, originating several diseases, such as acne, dandruff or atopic dermatitis, among others [20]. For instance, an increase in sebum by the sebaceous glands will trigger an increase of *C. acnes* and a decrease of *S. epidermidis* growth, resulting in a skin disorder known as acne [21]. Furthermore, the increased activity of the sebaceous glands can also induce the growth of several species of *Malassezia,* which can cause several pathologies, including seborrheic dermatitis, atopic dermatitis and psoriasis [22]. Thus, it is of utmost relevance to find compounds with antimicrobial properties that can target these microorganisms contributing to the treatment of the aforementioned pathologies in order to maintain skin homeostasis.

In recent decades, marine organisms have been targeted for their ability to produce compelling compounds with high bioactive potential. Seaweeds are a class of pluricellular organisms, visible to the naked eye, mostly sessile, capable of adapting to harsh ever-changing environmental conditions, by producing molecules such as proteins, amino acids, carbohydrates, pigments, vitamins and polyphenols as a defence mechanism. These classes of molecules have been previously described as potent antioxidant, antimicrobial or anti-inflammatory agents and, therefore, are prime targets for the development of new skincare formulations [23,24]. *Carpomitra costata* (Stackhouse) Batters 1902, a brown seaweed (Phaeophyceae), can be found throughout the Mediterranean Sea and North Atlantic Ocean, on the coast of Ireland and Portugal. This seaweed, yet poorly studied, has revealed antifungal, anti-inflammatory, antioxidant and photoprotective properties [25,26,27]. Therefore, the main objective of this study was to investigate the potential of *C. costata* as a source of bioactive compounds with dermatological potential by means of antioxidant, anti-enzymatic (collagenase, elastase, hyaluronidase and tyrosinase), antimicrobial (*S. epidermidis*, *C. acnes* and *M. furfur*), photoprotective and anti-inflammatory capacities for the development of natural-derived, safer, and effective skincare products.

## 2. Results

### 2.1. Extraction and Fractionation of Carpomitra costata

**Figure 1:**
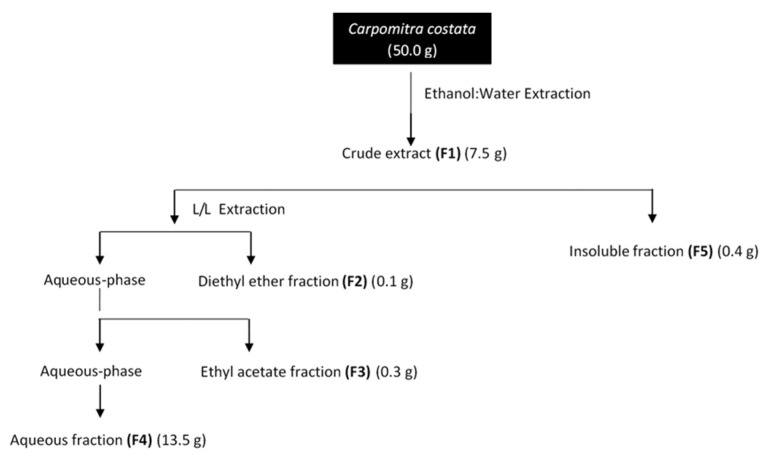
Extraction and fractionation flowchart of *Carpomitra costata*.

*Carpomitra costata* freeze-dried samples were subjected to an extraction and fractionation process resulting in five fractions (F1–F5) according to Figure 1.

### 2.2. Antioxidant Activity

The antioxidant potential of *C. costata* fractions was determined by three complementary assays, 2,2-diphenyl-1-picrylhydrazyl radical (DPPH) scavenging activity, ferric reducing antioxidant power (FRAP), and oxygen radical absorbance capacity (ORAC) and related to their total phenolic content (TPC). Results are presented in Table 1.

As shown in Table 1, the ethyl acetate fraction (F3) exhibited the highest phenolic content (321.3 ± 1.4 mg PE/g) and the greatest antioxidant potential, with the best capacity for scavenging DPPH radicals (EC_50_ of 140.1 µg/mL). This fraction also revealed the highest values of FRAP (474.6 ± 12.3 µM FeSO_4_/g) and ORAC (2082.4 ± 40.1 µmol TE/g). Both in the DPPH and ORAC assays, fraction F3 showed highest activity as compared to the antioxidant standard BHT. On the other hand, the aqueous fraction (F4) exhibited the lowest total phenolic content (12.9 ± 2.3 mg PE/g) and the lowest antioxidant potential in the three performed assays.

### 2.3. Enzymatic Inhibitory Activity

The results of the inhibitory effects of *C. costata* fractions on collagenase, elastase, hyaluronidase and tyrosinase activity are summarized in Table 2.

All fractions showed ability to inhibit one or more of the assayed enzymes. The ethyl acetate fraction (F3) exhibited the highest anti-enzymatic capacity, inhibiting the activity of collagenase (IC_50_ of 7.2 µg/mL), elastase (IC_50_ of 4.8 µg/mL) and tyrosinase (IC_50_ of 85.9 µg/mL). Additionally, the crude extract (F1), also revealed potential to inhibit the activity of collagenase (IC_50_ of 104.0 µg/mL), elastase (IC_50_ of 83.9 µg/mL) and hyaluronidase (IC_50_ of 47.4 µg/mL). However, fractions F2, F4 and F5 exhibited less anti-enzymatic activity, only inhibiting the activity of one enzyme, as shown in Table 2. Regarding collagenase, fractions F3 and F1 were the only ones that inhibited this enzyme but with a lower activity than the reference standard EGCG (IC_50_ of 4.8 µg/mL), while for elastase, both fractions showed greater inhibitory activity than this standard (IC_50_ of 113.9 µg/mL). The best hyaluronidase inhibitory ability was evidenced by fractions F1 (IC_50_ of 47.4 µg/mL), F2 (IC_50_ of 46.2 µg/mL) and F5 (IC_50_ of 48.1 µg/mL) as comparing with EGCG (IC_50_ of 119.1 µg/mL). Finally, for tyrosinase, only fraction F3 showed inhibitory capacity over this enzyme, however with less potency than the standard kojic acid (IC_50_ of 18.3 µg/mL).

### 2.4. Principal Components Analysis (PCA)

**Figure 2:**
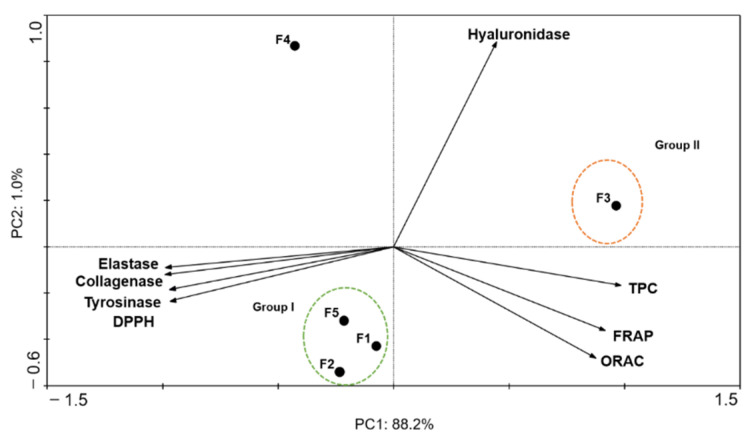
Principal component analysis (PCA) of antioxidant potential (TPC, DPPH, FRAP and ORAC), and enzymatic inhibitory activities (collagenase, elastase, hyaluronidase and tyrosinase) of *Carpomitra costata* fractions.

To correlate the antioxidant and anti-enzymatic activities of *C. costata* fractions, a PCA was performed, and the results are depicted in Figure 2.

The first two main components explain 88.2% and 1.0% of the total variance, respectively. The hyaluronidase presented a negative correlation with the other enzymes (collagenase, elastase and tyrosinase) and DPPH, and a null correlation with the antioxidant assays (TPC, FRAP and ORAC). On the other hand, antioxidant assays showed a positive correlation with each other, along with collagenase, elastase and tyrosinase. The ethyl acetate fraction (F3) presented the highest antioxidant capacity (Group II), with higher values of TPC, FRAP and ORAC and greater potential for DPPH scavenging (low EC_50_ values). F3 also revealed the highest anti-collagenase, anti-elastase and anti-tyrosinase activities. On the other hand, F1, F2 and F5 (Group I) demonstrated higher anti-hyaluronidase activity.

### 2.5. Antimicrobial Activity

**Figure 3:**
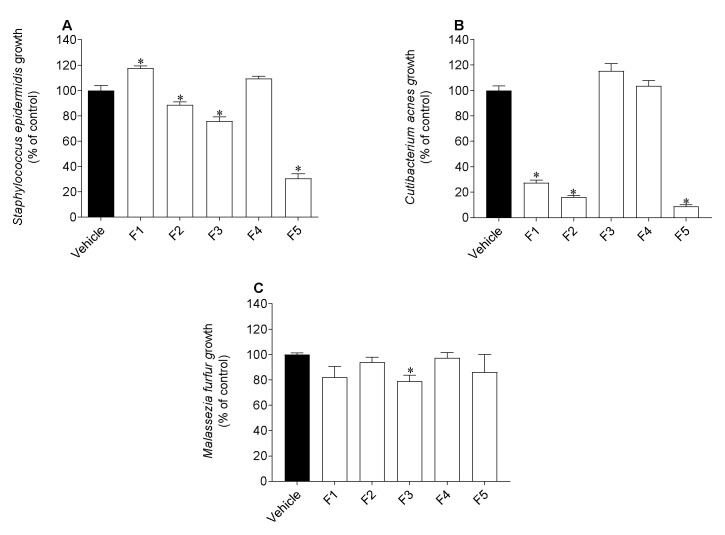
Antimicrobial activity of *Carpomitra costata* fractions (200 µg/mL) against *Staphylococcus epidermidis* (**A**), *Cutibacterium acnes* (**B**) and *Malassezia furfur* (**C**). The values correspond to mean ± SEM of three independent experiments. Symbol (*) represent significant differences (One-way ANOVA, Dunnett’s test; *p* < 0.05) when compared to vehicle.

The antimicrobial activity of the *C. costata* fractions was evaluated against two Gram positive bacteria, *Staphylococcus epidermidis* and *Cutibacterium acnes*, and one fungus, *Malassezia furfur*. Results are shown in Figure 3 and Table 3.

For the fractions that reduced microbial growth in more than 50%, their IC_50_ was determined and the results are presented in Table 3.

Generally, fractions influenced the growth of microorganisms and the less polar fraction (F5) showed the highest antimicrobial properties. Specifically, F5 revealed to be the fraction with the greatest inhibitory potential against *S. epidermidis* growth (IC_50_ of 72.0 µg/mL), followed by F3 and F2, which reduced its growth approximately by 20% and 10%, respectively (Figure 3). Regarding the growth of *C. acnes*, F2 showed the greatest inhibitory potential (IC_50_ of 45.9 µg/mL), followed by F5 (IC_50_ of 46.3 µg/mL) and F1 (IC_50_ of 141.4 µg/mL). None of the fractions showed a strong inhibition of *M. furfur* growth, however, fraction F3 reduced its growth in approximately 20% (Figure 3). From the obtained results, *C. costata* fractions prove to be more effective against bacteria, such as *S. epidermidis* and *C. acnes* than against the fungus *M. furfur*.

### 2.6. Biological Activities of Carpomitra costata Fractions on In Vitro Cellular Models

#### 2.6.1. Photoprotective Capacity in 3T3 Cells

**Figure 4:**
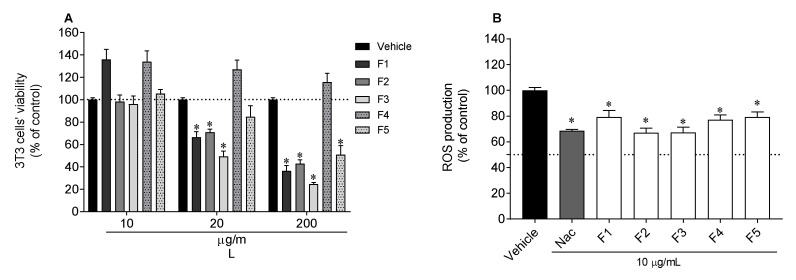
3T3 cells’ viability following 24 h of exposure to *Carpomitra costata* fractions (10; 20; 200 µg/mL) (**A**). Evaluation of reactive oxygen species (ROS) production by 3T3 cells exposed to UV radiation (12.5 mJ/cm^2^) for 1 h, in the presence/absence of *C. costata* fractions (10 µg/mL) and *N*-acetyl-l-cysteine (NAC, 10 µg/mL) (**B**). Results are expressed as % of the control. The values correspond to mean ± SEM of three independent experiments. * represents significant differences (One-way ANOVA, Dunnett’s test; *p* < 0.05) when compared to the vehicle.

Firstly, the viability of murine fibroblasts (3T3 cells) was evaluated when exposed to *C. costata* fractions (Figure 4A). It was observed that, at 200 and 20 µg/mL, several fractions were still toxic to the cells. At 10 µg/mL none of the fractions was found to be toxic and, therefore, this last concentration was selected to evaluate the photoprotective ability of *C. costata* fractions (Figure 4B). All fractions exhibited photoprotective capability, reducing the production of reactive oxygen species (ROS) stimulated by ultraviolet (UV)-exposure to values very close to those of the standard *N*-acetyl-l-cysteine (NAC) (Figure 4).

#### 2.6.2. Quantification of Nitric Oxide (NO) Produced by RAW 264.7 Cells

**Figure 5:**
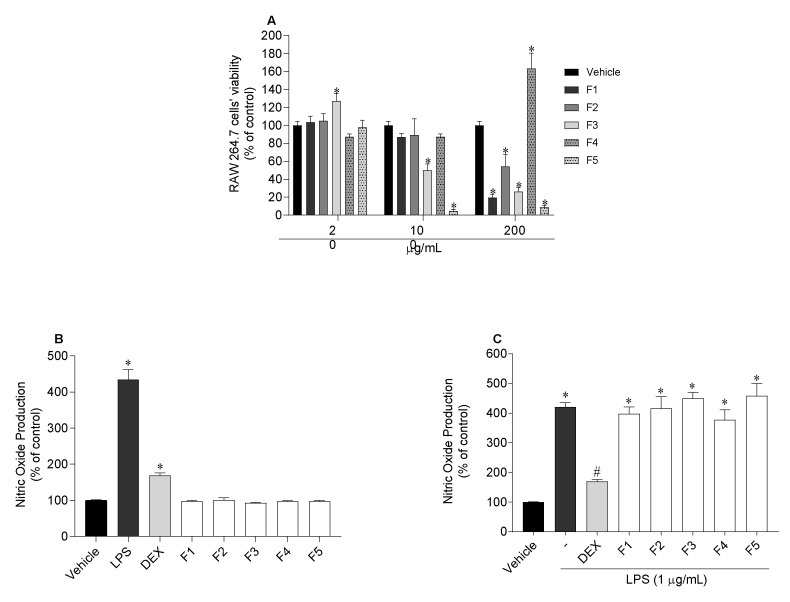
RAW 264.7 cells’ viability following 24 h of exposure to *Carpomitra costata* fractions (20; 100; 200 µg/mL) (**A**). Nitric oxide (NO) production by RAW 264.7 cells in the presence of *Carpomitra costata* fractions (20 µg/mL), LPS (1 µg/mL) and dexamethasone (DEX) (20 µg/mL) (**B**). NO production by RAW 264.7 exposed to LPS in the presence/absence of *Carpomitra costata* fractions (20 µg/mL) and DEX (20 µg/mL) (**C**). Results are expressed as % of the control. DEX was used as an anti-inflammatory standard. The values correspond to mean ± SEM of three independent experiments. Symbols represent significant differences (One-Way ANOVA, Dunnett’s test; *p* < 0.05) when compared to the vehicle (*) and LPS (^#^).

The viability of murine macrophage cells (RAW 264.7 cells) was evaluated when exposed to *C. costata* fractions to determine the non-toxic concentration (Figure 5A). At 20 µg/mL none of the fractions was found to be toxic to cells, being this concentration selected to quantify nitric oxide (NO) production of RAW 264.7 cells in normal and inflammatory conditions. The results are displayed in Figure 5. After RAW 264.7 cells’ exposure to seaweed fractions, it was observed that none stimulated the NO production (Figure 5B). On the other hand, none of the fractions showed an ability to decrease the NO levels induced by lipopolysaccharides (LPS) treatment (Figure 5C).

#### 2.6.3. Assessment of Inflammatory and Anti-Inflammatory Cytokines Levels

**Figure 6:**
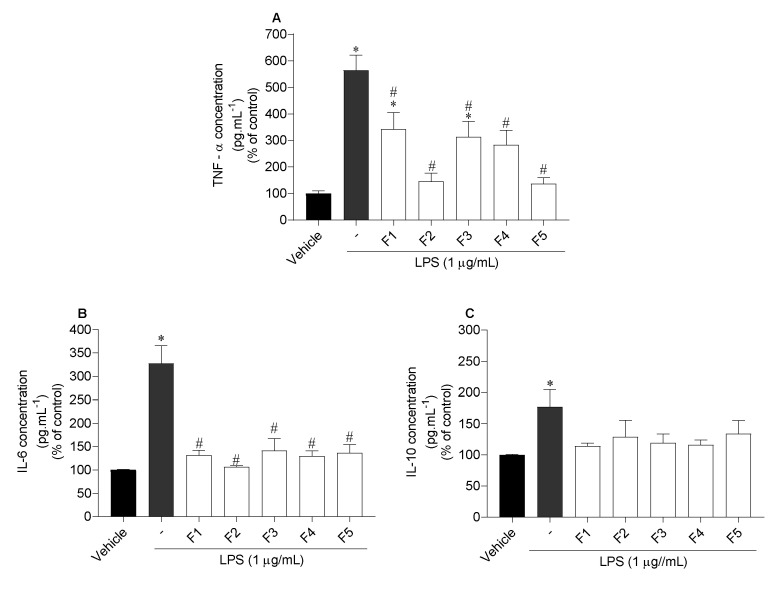
Effects of *Carpomitra costata* fractions (20 µg/mL) on the concentration levels of TNF-α (**A**), IL-6 (**B**) and IL-10 (**C**) cytokines in LPS exposed RAW 264.7 cells. The values correspond to mean ± SEM of three independent experiments. Symbols represent significant differences (One-way ANOVA, Dunnett’s test; *p* < 0.05) when compared to the vehicle (*) and LPS (^#^).

The release of the inflammatory cytokines tumour necrosis factor alpha (TNF-α), and interleukin 6 (IL-6), and anti-inflammatory interleukin 10 (IL-10) by RAW 264.7 cells exposed to LPS in the presence/absence of seaweed fractions was evaluated, and the results are displayed in Figure 6.

Regarding TNF-α, all fractions proved to be efficient in reducing the concentration of this cytokine, although F2 and F5 revealed the highest potential. When inflammation was induced by LPS, TNF-α concentration was 564.2 ± 54.9%. However, when the cells were treated with F2 and F5 fractions, TNF-α levels decreased to 145.5 ± 29.8% and 136.8 ± 21.7%, respectively (Figure 6A). Concerning IL-6, all fractions also showed to be efficient in reducing the levels of IL-6, however, F2 presented the greatest potential, decreasing the concentration from 327.5 ± 34.4% to 106.7 ± 2.0% (Figure 6B). On the other hand, none of them stimulated the release of IL-10 levels (Figure 6C).

### 2.7. Chemical Characterization of Carpomitra costata Fractions

#### 2.7.1. UV-VIS Absorption Spectra

**Figure 7:**
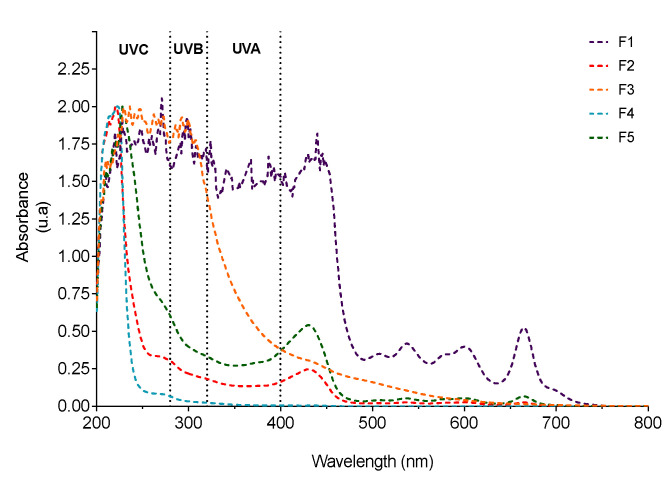
UV-VIS absorption spectra (200–800 nm) of *Carpomitra costata* fractions.

In order to correlate the photoprotective capacity of *C. costata* fractions with their chemical profile, the UV-VIS spectrum of each sample was acquired (Figure 7).

The crude extract (F1) exhibited characteristic absorption maximums of pigments such as chlorophylls (420–680 nm) and carotenoids (430–500 nm). Additionally, absorption peaks in the UVA (320–400 nm), UVB (280–320 nm) and UVC (200–280 nm) regions were also observed, confirming the complexity of its chemical composition. The most apolar fractions (F2 and F5) showed a very similar profile, with absorption maximums at 228 nm and 430 nm, probably due to the presence of fucoxanthin, a carotenoid commonly found in brown seaweeds. The ethyl acetate fraction (F3) has shown an absorption profile in the UVB (280–320 nm) and UVC (200–280 nm) ranges. Two absorption maxima at 271 and 262 nm suggest the presence of phlorotannins, a group of phenolic compounds almost exclusively found in brown seaweeds and reported for their strong photoprotective capacity. Finally, the most hydrophilic fraction (F4) only showed a maximum at 222 nm, restricting its photoprotective potential to the UVC zone.

#### 2.7.2. Nuclear Magnetic Resonance (NMR) Spectra

**Figure 8:**
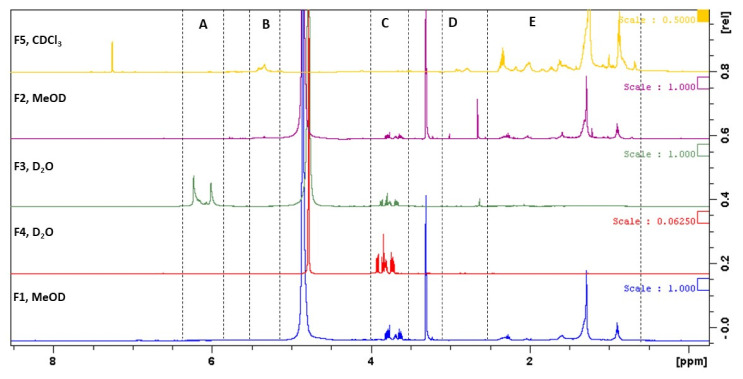
^1^H NMR (400 MHz) spectra of *Carpomitra costata* fractions.

The chemical profile of all fractions obtained from *C. costata* (F1–F5) was evaluated by ^1^H NMR and the corresponding spectra are depicted in Figure 8.

Despite the large variety of overlapping resonances, ^1^H NMR spectra of *C. costata* fractions were investigated based on the chemical shift assignments described in the literature for compounds frequently found in brown seaweeds. Accordingly, several regions of chemical shifts were considered in each spectrum (Figure 8, zones A–E).

The crude extract (F1) evidenced signals in the region of 0.88–2.36 ppm (Figure 8, zone E), characteristic of the most apolar compounds, like fatty acids, sterols and other lipids, including pigments [28,29,30]. In this fraction, signals in the range of 3.61–3.81 ppm (Figure 8, zone C) were also observed, which can be attributed to mannitol, a common polyol present in brown seaweeds [29,31,32,33]. A very similar profile was observed in the diethyl ether fraction (F2), although signals attributed to mannitol were not so intense. On the other hand, an intense peak at 2.65 ppm (Figure 8, zone D) suggests the presence of protons bound to carbon atoms in the alpha position to unsaturated groups in allylic, carbonyl, or imino groups, or protons of amines, which resonate in the range of 1.8–3.2 ppm [29,34]. Concerning the ethyl acetate fraction (F3), signals in the range of 5.92–6.13 ppm (Figure 8, zone A) can be attributed to the aromatic protons of phlorotannins, a group of phenolic compounds very common in brown seaweeds [31,35]. Within this group of compounds, signals of phloroglucinol (5.78 ppm), phloretol (5.92 ppm) and fucophloretol (6.10 ppm) were previously reported [35]. The presence of mannitol (Figure 8, zone C) in this fraction was also detected, although the highest amounts of this polyol were present in the aqueous fraction (F4) as evidenced in Figure 8. Finally, the non-soluble water fraction (F5) pinpoints its richness in most apolar molecules as evidenced by the signals in the range of 0.68–2.37 ppm (Figure 8, zone C) [28,29,30] having some of these compounds’ double bonds due to the existence of several olefinic signals between 5.2 and 5.5 ppm (Figure 8, zone B).

## 3. Discussion

Skin is the largest organ in the human body and plays an important role in the defence of the organism, acting as a protective barrier against external stimuli, like pollution particles, microorganisms, chemicals, and UV radiation [36,37]. Increased ROS and consequent oxidative stress play a major role in skin aging, being involved in the development of several skin disorders [38,39]. Thus, managing ROS production and decreasing their damaging effects may be the key to prevent and treat various skin disorders. In recent years, brown algae have been the subject of several studies regarding their cosmetic applicability [40,41]. It is well known that seaweeds are a source of a vast class of high bioactive molecules that can act as strong antioxidant agents [42], improving cosmetic formulations by protecting skin from ROS-induced damage [23].

In the present work, *C. costata* was subjected to sequential extractions, compatible with cosmetic applications, affording five fractions which chemical profiles were attained by NMR and UV-VIS spectroscopy. All of them were tested for their antioxidant ability and total phenolic content. Through an integrative analysis of the results obtained in the antioxidant-related assays, the ethyl acetate fraction (F3) stood out for its high radical scavenging capacity, which seems to be closely associated with its high phenolic content (321.3 ± 1.4 mg PE/g extract). Obluchinskava and co-workers [43] also reported the phlorotannin content of two brown algae, *Fucus vesiculosus* (174.8 ± 2.0 mg/g extract) and *Ascophyllum nodosum* (148.0 ± 1.6 mg/g extract). The low values observed in the phenolic content of those seaweeds, when compared with *C. costata,* can be influenced by the seasonality of the species but also by the use of different solvents and extraction methodologies that can affect significantly the extraction of phenolic compounds from natural matrices. Furthermore, in a study by Lim and co-workers [44], the phenolic content of the brown seaweed *Sargassum serratifolium* was also evaluated, using different extraction solvents. These results showed that solvent selection is important for the recovery of phenolic compounds, and that ethyl acetate afforded the highest phenolic content (105.0 ± 2.4 mg/g extract), which is in agreement with results here presented. However, the phenolic content of *S. serratifolium* is low when compared to *C. costata*.

Effectively, the performed extraction process has conducted to an enriched phenolic fraction (F3), which NMR spectra evidenced signals of phlorotannins [31,35]. These compounds are produced exclusively by brown algae, and have been associated with a strong antioxidant, photoprotective and antimicrobial capacity [40,45,46,47]. Some studies have evaluated the antioxidant activity of seaweeds. In a study by Babaei and co-workers [48], the antioxidant activity of two brown seaweeds was evaluated, revealing that the ethyl acetate fractions showed the best results, due to their high phenolic content, including phlorotannins. The results obtained in this work are in accordance with those described in the literature, where *C. costata* is reported to possess a strong antioxidant potential [26].

The direct exposure of the skin to sunlight is considered the main factor for photoaging, being responsible for the reduction of ECM components, such as collagen, elastin, and hyaluronic acid (HA) [49]. These components are essential to maintain a healthy skin and, therefore, the inhibition of enzymes associated with their degradation, such as collagenase, elastase, and hyaluronidase, is an effective approach to prevent skin aging. Moreover, the inhibition of tyrosinase is also important to prevent undesired excessive skin pigmentation. In this study, the inhibitory effects of *C. costata* fractions on collagenase, elastase, hyaluronidase and tyrosinase were evaluated. The results showed that fraction F3 presented the highest inhibitory activity on all studied enzymes, except on hyaluronidase, revealing to be more potent in inhibiting elastase than the standard compound epigallocatechin gallate, widely known for its skin benefits [50]. This strong inhibitory potential may be associated with a high content in phenolic compounds, namely phlorotannins, since these compounds have already reported to have high antioxidant, photoprotective and anti-enzymatic properties [23,40,51,52,53]. Although not so expressive as fraction F3, the crude extract (F1) also showed a strong inhibitory ability of collagenase, elastase and hyaluronidase, which, due to its low antioxidant expression, can be related with non-phenolic compounds, e.g., pigments, namely fucoxanthin, terpenes and sulphated polysaccharides, among others, that can be extracted from seaweeds with hydroalcoholic mixtures [23]. In a study performed by Rui and co-workers [54], mycosporine-like amino acids (MAAs) extracted from the macroalgae *Porphyra tenera*, inhibited the expression of collagenase and another extracellular matrix related MMPs. Moreover, sulphated polysaccharides have been previously described as being able to suppress tyrosinase activity [55].

A principal component analysis (PCA) was performed in order to further assess if a relationship between antioxidant and anti-enzymatic activity exists. This analysis showed that there is a strong correlation between the antioxidant potential and extracellular matrix (ECM) enzymes inhibition mediated by fraction F3. Through this analysis, it was also possible to verify that fractions F1, F2 and F5 exhibited a high capacity to inhibit hyaluronidase, suggesting that the most apolar compounds [28,30] present in these fractions can exert some inhibition over this enzyme. On the contrary, mannitol [31,32,33], the major component of fraction F4, seems to have no influence neither in antioxidant nor in anti-enzymatic properties.

As it is well known, the skin microbiota plays a vital role in the maintenance of a healthy skin. Thus, one of the most important steps towards the development of new cosmetic formulations consists in the evaluation of new compounds’ effects against skin microbiota. The three microorganisms selected for this study, *S. epidermidis*, *C. acnes* and *M. furfur* are part of the skin’s natural microbiome and generally considered commensals, as they are harmless and benefit the skin in healthy conditions [40]. However, disturbance in the skin barrier leads to the formation of biofilms by *S. epidermidis* and *C. acnes*, which can originate several diseases, such as atopic dermatitis and acne, respectively [56,57]. Additionally, *M. furfur* is also associated with several skin disorders like pityriasis versicolor, folliculitis, seborrheic dermatitis, dandruff, atopic dermatitis, and psoriasis [22]. Regarding *S. epidermidis* and *C. acnes*, fraction F5 demonstrated a stronger growth inhibitory effect than the remaining fractions. This inhibition may be linked to the lipophilic compounds present in this fraction, as supported by other studies. In a study by Chen and co-workers [58] the potential of lipophilic compounds present in the essential oils of a pine species was evaluated, showing their ability to inhibit the growth of several microorganisms, such as Gram-positive bacteria. On the other hand, F3 revealed the potential to inhibit both *S. epidermidis* and *M. furfur*. This may be associated with the presence of phlorotannins and, eventually, other phenolic compounds, since they have already been described for their strong antimicrobial activities [40,59].

The exposure of human skin to ultraviolet radiation (UVR) promotes serious skin injuries, such as sunburn, blemishes, inflammation, photoaging and skin cancer [60,61]. Accordingly, there is a growing interest in the development of sunscreen products from natural biosynthesis instead of the commonly used synthetic products [62]. As seaweeds are usually attached to a substrate and, thus, periodically exposed to UVR, they developed a protective mechanism through the biosynthesis of photoprotective substances such as mycosporine-like amino acids, sulphated polysaccharides, pigments, and phenolic compounds, that neutralize and minimize radiation damage [23,53,63,64].

To evaluate the photoprotective potential of *C. costata* fractions, 3T3 cells, were exposed to UVA and UVB radiation, and ROS levels determined. Firstly, the cytotoxicity of each fraction was studied in order to define the sub-toxic concentrations. At 10 µg/mL, all fractions evidenced no cytotoxicity and showed a photoprotective effect on 3T3 cells when subjected to UVR, although this effect was more pronounced with fractions F2 and F3. This may be explained by its rich composition in photoprotective compounds, such as phlorotannins and, eventually, mycosporine-like amino acids (MAAs). Phlorotannins are known for their antioxidant and photoprotective capabilities since, due to the chromophores present in their structures, they are capable of neutralizing the harmful effects of oxidative stress after sun exposure [40,65]. In a study by El Aanachi and co-workers [66], the antioxidant and photoprotective potential of extracts enriched in phenolic compounds was evaluated, revealing their strong effect, due to their absorption capacity at wavelengths from 280 to 320 nm. On the other hand, Coba and co-workers [67] demonstrated that MAAs can be used as natural and safe compounds, presenting antioxidant and photoprotective effects, with potential against the harmful effects of UV radiation. In addition to F2 and F3, all remaining fractions showed a low photoprotective potential, slightly inhibiting the production of ROS. This may be due to the residual presence of compounds capable of protecting against damage caused by UVR [68,69]. These results are corroborated by the study already developed on the photoprotective activity of *C. costata,* which demonstrated that *C. costata* reduced the production of ROS by HaCaT cells, when stimulated by UVB radiation and decreased oxidative stress in DNA, lipids and proteins. These results support the potential of *C. costata* to be used in new formulations as a natural antioxidant, acting in the prevention of skin aging [26].

Inflammation is the first defensive reaction in response to different stimuli, such as infections, oxidative damage and external substances, among others [70]. However, inadequate inflammatory responses are responsible for the appearance of numerous chronic inflammatory diseases like irritable bowel syndrome, diabetes, cancer [71] and for the development and progression of skin diseases [10]. In the inflammatory process, TNF-α is initially released, accelerating the migration of neutrophils to the damaged areas, resulting in the excessive release of ROS [39] and, consequently, the development of inflammatory diseases. At the same time, IL-6 accelerates the release of other inflammatory mediators. Therefore, for the treatment of inflammatory diseases, it is important to find compounds with high antioxidant capacity against ROS, and with the potential to inhibit the expression of pro-inflammatory cytokines [72]. Several studies reported the potential of seaweeds in reducing inflammatory mediators, such as NO, TNF-α, IL-6 and IL-10 [73]. Thus, the anti-inflammatory potential of *C. costata* fractions was evaluated for their ability to inhibit the production of NO, TNF-α, IL-6, and to promote the expression of IL-10 in murine macrophage cells (RAW 264.7) stimulated by LPS. Firstly, the cytotoxicity of each fraction was studied in order to define the non-toxic concentrations (20 µg/mL) for the cells. It was possible to observe that the levels of NO were very high with the treatment with LPS, which means that the LPS induced cells to produce NO. None of the *C. costata* fractions promoted the production of NO, meaning that none of them induced an inflammatory status in the cells. On the other hand, none of the fractions had the potential to inhibit NO production. These results are not in accordance with a study already carried out with *C. costata* in which a high potential to inhibit NO production was observed [25]. Nevertheless, it should be noted that when working with natural biomass, there are several factors that can affect the bioactivities, e.g., the time and place of harvesting, the different extraction methodologies, solvents, and tested concentrations, among others.

Although the results regarding the ability to inhibit nitric oxide production have not been promising, it is known that there are different signalling pathways and, in this sense, other inflammatory and anti-inflammatory mediators such as TNF-α, IL-6 and IL-10 were also evaluated. Regarding these mediators, TNF-α plays a pro-inflammatory role in the biological system, IL-6 can promote the proliferation and differentiation of B cells and T cells and, lastly, IL-10, an anti-inflammatory cytokine with immunosuppressive effect [74,75]. Analysing the results here obtained, it is possible to observe that the levels of TNF-α, IL-6 and IL-10 were higher in the treatment with LPS than when compared to the vehicle, meaning that LPS induced the cells to release inflammatory cytokines. In general, it can be said that the diethyl ether fraction (F2) and the non-soluble water fraction (F5), which appear to be rich in more lipophilic compounds, had the greatest anti-inflammatory potential, reducing TNF-α and IL-6 levels, and showing no ability to stimulate IL-10 expression. However, both in TNF-α and IL-6, all fractions showed the potential to inhibit these cytokines.

These results are consistent with previously developed studies [25] in which the ethanolic extract of *C. costata* demonstrated anti-inflammatory potential, having the ability to inhibit inflammatory mediators, such as prostaglandin E_2_ (PGE_2_) and nitric oxide, by inhibiting iNOS and COX-2 in RAW 264.7 cells induced by LPS. Yim and co-workers [25] also demonstrated the potential of this extract to inhibit the expression of IL-1, TNF-α and IL-6, reducing the expression of transcription factors, nuclear factor-κB (NF-κB) and activator protein 1 (AP-1), suggesting its use in the treatment of inflammatory diseases. Due to the potential of *C. costata* to reduce the levels of TNF-α and IL-6, it would be pertinent to evaluate other key inflammatory mediators, such as COX, iNOS, PGE2, NF-κB, and IL-1β in order to deeply characterize the anti-inflammatory activity of those fractions. Furthermore, the study of IL-1β is particularly relevant because its expression increasing is directly correlated with exposure to UVB radiation and skin aging, through the expression of MMPs, namely MMP-1 and MMP-13. Furthermore, in inflammatory diseases such as psoriasis and atopic dermatitis, the increased expression of this cytokine is characterized by the disruption of the skin barrier [76]. Therefore, in future studies, it would be important to evaluate the expression of IL-1β due to its association with aging and skin pathologies.

In conclusion, among the samples here studied, the enriched polyphenolic fraction F3, due to its wide range of bioactivities as antioxidant, anti-enzymatic, antimicrobial, photoprotective, and anti-inflammatory capacity, may be suitable for further application in new skincare formulations. In addition to F3, the F5 fraction also revealed potential for application in new skin products, showing antimicrobial and anti-inflammatory activities. However, additional chemical and biological studies in more complex in vitro and in vivo models are needed to validate the results here obtained aiming the exploitation of *C. costata* as a source of natural ingredients for dermatological applications.

## 4. Materials and Methods

### 4.1. Seaweed Collection and Preparation

The brown seaweed *Carpomitra costata* (Stackhouse) Batters 1902 was collected off Berlenga Nature Reserve (39°24’55’’ N 9°30’34’’ O), Peniche (Portugal) in October 2018, and immediately transported to the laboratory, where it was identified by Prof. Teresa Mouga, a biologist with vast experience in taxonomic identification and ecology of marine seaweeds. After cleaning and washing with seawater to remove invertebrate organisms, epiphytes and debris, *C. costata* was frozen at −20 °C and freeze-dried (Scanvac Cool Safe, LaboGene, Lynge, Denmark). The dry algal material was ground into a powder in a grinder, and stored at room temperature, protected from light, until extraction procedures.

### 4.2. Seaweed Extraction

Freeze-dried samples of *C. costata* (50 g) were extracted at room temperature with ethanol: water (70:30, *v*/*v*, 1000 mL), at constant stirring, overnight, in the dark. The resulting liquid extract was evaporated until dryness to give the crude extract corresponding to the total hydro-alcoholic fraction (F1). Then, this fraction was re-suspended in hot (70 °C) water (100 mL), cooled, and filtered with quality paper No. 4 (VWR International, Alfragide, Portugal) giving a solid insoluble phase retained in the filter (F5) and a liquid (L) aqueous fraction. The last one was subjected to a L/L partition, firstly with diethyl ether (6 × 100 mL) and then, with ethyl acetate (10 × 100 mL). Organic phases were concentrated to dryness resulting in the diethyl ether (F2), ethyl acetate (F3) and aqueous (F4) fractions. The used solvents (*p.a*) were purchased from VWR-BDH Chemicals-Prolabo (Leuven, Belgium). All extracts were concentrated under reduced pressure, at low temperature (40 °C), in a rotary evaporator (IKA HB10, VWR International, Alfragide, Portugal) and/or in a speed-vacuum equipment (Eppendorf Concentrator Plus, Leicestershire, UK), while the remaining aqueous extract was freeze-dried. For bioassays, samples were dissolved in dimethyl sulfoxide (DMSO) at a concentration of 20 mg/mL. The extraction process of *C. costata* biomass is illustrated in Figure 1.

### 4.3. Evaluation of the Biological Activities of Carpomitra costata

#### 4.3.1. Antioxidant Activity

The antioxidant potential of *C. costata* fractions and of the antioxidant standard 3,5-di-*tert*-4-butylhydroxytoluene (BHT) was evaluated through three different methods, namely: 2,2-diphenyl-1-picrylhydrazyl (DPPH) radical scavenging activity, ferric reducing antioxidant power (FRAP) and oxygen radical absorbance capacity (ORAC). Additionally, the total phenolic content (TPC) of each sample was also quantified aiming to establish a relationship of this parameter with the antioxidant capacity.

I. Quantification of Total Phenolic Content (TPC)

TPC was determined by the Folin-Ciocalteu method [77], with slight modifications [40]. This method is based on the colorimetric reaction of phenolic substances with Folin-Ciocalteu reagent. After 1 h of reaction in the dark, the absorbance was measured at 750 nm in a microplate reader (Epoch Microplate Reader, BioTek^®^ Instruments, Winooski, VT, USA). Phloroglucinol was used as standard for the calibration curve, and TPC was expressed in milligrams of phloroglucinol equivalents per gram of dry extract (mg PE/g of extract).

II. 2,2 Diphenyl-1-picrylhydrazyl (DPPH) Radical Scavenging Activity

The capacity of *C. costata* fractions (200 µg/mL) to scavenge the DPPH radical was performed according to Brand-Williams and co-workers [78]. The reaction occurred for 30 min in the dark, and absorbance was measured at 517 nm in a microplate reader. For samples (10–200 µg/mL) with the capacity to scavenge the DPPH radicals greater than 50%, the EC_50_ was calculated.

III. Ferric Reducing Antioxidant Power (FRAP)

The FRAP assay was performed as described by Benzie and Strain [79], adapted to microscale with slight modifications [40]. FRAP reagent was prepared with 0.3 M acetate buffer (pH 3.6), 10 mM of 2,4,6-Tris (2-pyridyl)-*s*-triazine (TPTZ) in 40 mM HCl and 20 mM ferric solution using FeCl_3_ at a ratio of 10:1:1 and incubated at 37 °C. *C. costata* fractions were added to FRAP reagent and incubated in the dark for 30 min, at 37 °C, and the absorbance was measured at 593 nm in a microplate reader. FeSO_4_ was used as the standard for the calibration curve, and the results were expressed as micromolar of FeSO_4_ equivalents per gram of dry extract (µM of FeSO_4_/g of extract).

IV. Oxygen Radical Absorbance Capacity (ORAC)

The ORAC assay was performed according to Dávalos and co-workers [80]. Seaweed fractions were pre-incubated with fluorescein (70 nM) for 15 min at 37 °C. After this time, 2,2′-azobis (2-methylpropionamidine) dihydrochloride (AAPH) solution (12 mM) was added and the fluorescence (λ excitation: 458 nm; λ emission: 520 nm) was recorded every minute for 240 min in the microplate reader (Multimodal Synergy H1, BioTek^®^ Instruments, Winooski, VT, USA). Trolox was used as standard antioxidant, and the results were expressed as micromoles of Trolox equivalents per gram of dry extract (µmol TE/g of extract).

#### 4.3.2. Enzymatic Inhibitory Activity

The inhibitory effects of *C. costata* fractions on the activity of collagenase (type IV), elastase, hyaluronidase and tyrosinase enzymes were evaluated as follows described:

I. Anti-Collagenase Activity

The anti-collagenase activity was determined using the EnzChek™ Gelatinase/Collagenase Assay Kit (# E12055, Invitrogen™, ThermoFisher Scientific) according to manufacturer’s instructions. Epigallocatechin gallate (EGCG) was used as positive control and the results were expressed as arbitrary fluorescence units per minute (∆ fluorescence (a.u.)/min) as percentage of the control. To the fractions with the greatest potential to inhibit collagenase (> 50%), a dose-response (100, 60, 30, 10 µg/mL) analysis was conducted and the IC_50_ was determined.

II. Anti-Elastase Activity

The anti-elastase activity was determined using the EnzChek™ Elastase Assay Kit (# E12056, Invitrogen™, ThermoFisher Scientific) according to manufacturer’s instructions. EGCG was used as positive control and the results were expressed as arbitrary fluorescence units per minute (∆ fluorescence (a.u.)/min) as percentage of the control. Fractions with the greatest potential to inhibit elastase (> 50%) were subjected to a dose-response analysis (100, 60, 30, 10 µg/mL) and the IC_50_ was determined.

III. Anti-Hyaluronidase Activity

The inhibition of hyaluronidase activity was determined following the method described by Yahaya and Don [81] with slight modifications and adapted to the microscale [40]. Briefly, 3 µL of each fraction was mixed with 5 µL of hyaluronidase (7 U/mL) and 67 µL of enzyme diluent (20 mM sodium phosphate, 77 mM sodium chloride and 0.01% bovine serum albumin (BSA); pH 7.0 at 37 °C) and pre-incubated at 37 °C for 10 min. After that, 25 µL of hyaluronic acid solution (0.03% in 300 mM sodium phosphate; pH 5.35 at 37 °C) were added and incubated for 45 min at 37 °C. Hyaluronic acid was then precipitated using 200 µL of acidic albumin solution (24 mM sodium acetate, 79 mM acetic acid and 0.1% BSA; pH 3.75 at 25 °C). After 10 min at room temperature, the absorbance was measured at 600 nm. The absorbance in the absence of enzyme was used as the control value for maximum inhibition. The hyaluronidase inhibitory activity of each fraction was determined as:

(1) $$\mathrm{Hyaluronidase}\;\mathrm{inhibitory}\;\mathrm{activity}\;=\;\frac{Ab_{sample}\;-\;Ab_{blank}}{Ab_{control}}$$

where *Abs_sample_* is the absorbance of sample with hyaluronidase, hyaluronic acid, and acidic albumin, *Abs_blank_* is the absorbance of sample, hyaluronidase, and acidic albumin, and *Abs_control_* is the absorbance of hyaluronic acid and acidic albumin (without hyaluronidase). Fractions that inhibit the hyaluronidase activity (>50%) were additionally tested at 100, 60, 30 and 10 µg/mL, and the IC_50_ was determined.

IV. Anti-Tyrosinase Activity

The inhibition of tyrosinase activity was performed as described by Senol and co-workers [82] and Lee and co-workers [83], with slight modifications. This method is based on the oxidation of l-3,4-dihydroxyphenylalanine (L-DOPA) by the tyrosinase. Briefly, 2 µL of each fraction was mixed with 68 µL of potassium phosphate buffer (0.5 mM, pH 6.8) and 100 µL of L-DOPA (1 mM) and pre-incubated at 37 °C for 5 min in the dark. After the pre-incubation time, 30 µL of tyrosinase (100 U/mL) were added and the absorbance was measured at 475 nm, and every minute thereafter for 15 min, in the microplate reader. Kojic acid was used as standard, and the results were expressed as a percentage of control.

#### 4.3.3. Antimicrobial Activity

Antimicrobial activity of *C. costata* fractions was evaluated against three different microorganisms, as previously described [40]. Two Gram-positive bacteria, *Staphylococcus epidermidis* (DSM 1798) and *Cutibacterium acnes* (DSM 1897), and one fungus, *Malassezia furfur* (DSM 6170) were acquired from Leibniz Institute DSMZ-German Collection of Microorganisms and Cell Cultures (DSMZ) biobank. Briefly, *S. epidermidis* was grown at 37 °C, on trypticase soy broth medium, *C. acnes* at 37 °C, on Tryptic Soy Broth with anaerobic conditions media, and *M. furfur* at 30 °C on Leeming-Notman medium. The antimicrobial activity of each fraction (200 µg/mL) was determined during the exponential growth, at 600 nm. Oxytetracycline was used as a positive control of *S. epidermidis* and *C. acnes*, and amphotericin B for *M. furfur*. Results were expressed as percentage of control. For the fractions with the highest potential (>50% inhibition of the microorganism) a dose-response analysis (100, 60, 30 and 10 µg/mL) was performed and the IC_50_ values determined.

### 4.4. Biological Activity of Carpomitra costata Fractions on in Vitro Cellular Models

The cytotoxic, photoprotective, inflammatory and anti-inflammatory activities of *C. costata* fractions were evaluated on different cellular models as described below.

#### 4.4.1. Cell Culture Maintenance

Fibroblast cells from Swiss albino mouse embryo tissue (3T3-ACC-173) and mouse macrophage cells (RAW 264.7 -ATCC-TIB-71) were acquired from the DSMZ and American Type Culture Collection (ATCC) biobanks, respectively. The 3T3 cells were cultured in DMEM F12 (Dulbecco’s Modified Eagle’s medium: Nutrient Mix F12) supplemented with 10% fetal bovine serum (FBS), 100 IU/mL penicillin, and 100 µg/mL streptomycin. The RAW 264.7 cells were cultured in DMEM without phenol red supplemented with 10% fetal bovine serum (FBS), 1% antibiotic/antimycotic (Amphotericin B, Penicillin and Streptomycin) and 1% sodium pyruvate. Cells were kept in a 95% moisture and 5% CO_2_ atmosphere at 37 °C. Subculture was performed according to biobank instructions whenever cultures reached 80–85% confluence.

#### 4.4.2. Cytotoxicity Evaluation

The cytotoxic activities of *C. costata* fractions were evaluated by the 3-[4, 5-dimethylthiazol-2-yl]-2, 5-diphenyltetrazolium bromide (MTT) colorimetric assay, as previously described by Yuan and Walsh [84]. Firstly, 3T3 and RAW 264.7 cells were seeded in 96-well plates, at a density of 5 × 10^4^ cells/well and incubated until they reached total confluence. Cells were then treated with the seaweed fractions (200 µg/mL) for 24 h. After this time, 100 µL of MTT solution was added to all wells, and the microplates incubated at 37 °C for 1 h. MTT was then discarded, the formazan crystals solubilized with dimethyl sulfoxide (DMSO), and the absorbance measured at 570 nm (Epoch Microplate Reader, BioTek^®^ Instruments, Winooski, VT, USA). Untreated cells were used as a control. Saponin was used as a positive control for cellular death. The results were expressed as a percentage of control.

#### 4.4.3. Photoprotective Capacity in 3T3 Cells

The capacity of the seaweed fractions to reduce the ROS production was performed as described by Marto and co-workers, with slight modifications [85]. Cells were treated with *C. costata* fractions at non-toxic concentrations (10 µg/mL) for 1 h, at 37 °C, in the dark. Treated cells were then exposed to UV radiation (12.5 mJ/cm^2^) for 1 h, in a UV curing chamber (UVA Cube 400, Hönle Technology, Gräfelfing, Germany). Later, 100 µL of 2’,7’-dichlorodihydrofluorescein diacetate (H2-DCFDA) (20 µM) was added to cells, which were then incubated for 30 min, at 37 °C, in the dark. ROS levels were determined by measuring fluorescence (λ excitation: 495 nm; λ emission: 527 nm) every minute, for 10 min. The results were expressed as a percentage of control.

#### 4.4.4. Quantification of Nitric Oxide on Mouse Macrophage Cells

The inflammatory and anti-inflammatory effects of *C. costata* fractions were determined by the nitric oxide (NO) production as described by Yang and co-workers [86] with slight modifications. Firstly, cells were seeded in 96-well plates (5 × 10^4^ cells/well) and incubated for 16 h. Cells were treated with fractions at a non-toxic concentration (20 µg/mL) for 24 h, to evaluate the production of NO. The anti-inflammatory effects of *C. costata* fractions were then evaluated by exposing cells to *C. costata* fractions for 1 h, prior to a 24 h incubation with lipopolysaccharide (LPS) as inflammation mediator, at 1 µg/mL. Then, 150 µL of culture medium from each well were transferred to a new plate, and 50 µL of Griess reagent (1% (*w/v*) sulphanilamide, 0.1% (*w*/*v*) *N*-(1-naphthyl) ethylenediamine in 2.5% (*v*/*v*) phosphoric acid) was added. The mixture was incubated for 30 min, at room temperature, in the dark, and absorbance was measured at 546 nm. Dexamethasone (DEX) was used as positive control. The results were expressed as percentage of control untreated cells.

#### 4.4.5. Effects of *Carpomitra costata* Fractions on Inflammatory and Anti-inflammatory Cytokine Mediators

The effect of *C. costata* fractions on the inflammatory (TNF-α and IL-6) and anti-inflammatory (IL-10) mediators was evaluated using the ELISA assay in RAW 264.7 cells treated with LPS. Cells were seeded in 12-well plates (5 × 10^5^ cells/well) and incubated for 18 h with *C. costata* fractions and LPS. The supernatant (1000 µL) was collected and the levels of TNF-α, IL-6 and IL-10 were quantified using the TNF Alpha Mouse uncoated ELISA Kit (# 88-7324-22, Invitrogen™, ThermoFisher Scientific, Waltham, MA, USA), IL-6 Ready-SET Go ELISA Kit (#88-7064-22, Invitrogen™, ThermoFisher Scientific) and IL-10 Mouse uncoated ELISA Kit (#88-7105-22, Invitrogen™, ThermoFisher Scientific), respectively, according to the manufacturer’s instructions. The absorbance was measured at 570 nm and 450 nm, to allow subtraction of the wavelength. TNF-α, IL-6 and IL-10 levels were expressed as a percentage of the control.

### 4.5. Chemical Characterization of Carpomitra costata Fractions

The chemical profile of *C. costata* fractions (F1–F5) was attained by ultraviolet-visible (UV-VIS) spectroscopy and nuclear magnetic resonance (NMR) spectroscopy.

#### 4.5.1. UV-VIS Spectroscopy Analysis

Samples were dissolved in dichloromethane or methanol (1 mg/mL) and their UV-VIS absorption spectra obtained on a UV-VIS spectrophotometer (Evolution 201, Thermo Scientific, Madison, WI, USA) in the wavelength range of 200–800 nm.

#### 4.5.2. NMR Spectroscopy Analysis

For NMR analysis, the samples (*c.a* 5–6 mg) were dissolved in 0.5 mL of deuterated solvents (CDCl_3_, MeOD, or D_2_O; Sigma-Aldrich, St. Louis, MO, USA) and the ^1^H NMR spectra were recorded at 400.13 MHz on a Bruker AMX400 spectrometer, at 25 °C. Chemical shifts (δ) are expressed in ppm and referenced to the residual solvent signal (δ_H_ = 7.26 ppm, CDCl_3_; δ_H_ = 3.31 ppm, MeOD; δ_H_ = 4.79 ppm, D_2_O).

### 4.6. Data and Statistical Analysis

The significance of the differences between samples and controls were determined using one-way analysis of variance (ANOVA) with Dunnett’s multiple comparison tests. All data were checked for normality (Shapiro-wilk test) and homoscedasticity (Levene’s test). The EC_50_/IC_50_ was determined using the software GraphPad v5.1 by means of the equation y = 100/(1 + 10 (X − Log IC_50_)). The differences were considered significant at a level of 0.05 (*p <* 0.05). Principal component analysis (PCA) was performed with CANOCO for Windows 4.5 software. Calculations were performed using IBM SPSS Statistics 24 (IBM Corporation, Armonk, NY, USA) and GraphPad v5.1 (GraphPad Software, La Jolla, CA, USA) software. All data were obtained from at least three independent experiments carried out with four replicates. The results are presented as the mean ± standard error of the mean (SEM).

## Figures and Tables

**Table 1:** Antioxidant capacity of *Carpomitra costata* fractions and BHT.

Fraction	TPC ^a^	DPPH ^b^	FRAP ^c^	ORAC ^d^
F1	43.9 ± 1.1	>200	158.3 ± 21.8	729.1 ± 8.8
F2	31.8 ± 20.7	>200	171.8 ± 50.1	696.8 ± 179.3
F3	321.3 ± 1.4	140.1 (106.2–186.0)	474.6 ± 12.3	2082.4 ± 40.1
F4	12.9 ± 2.3	>200	62.5 ± 22.3	207.2 ± 27.2
F5	29.9 ± 0.4	>200	122.4 ± 6.7	640.6 ± 5.7
BHT	-	164.5 (142.7–189.7)	2821.5 ± 51.5	142.9 ± 9.1

^a^ mg of phloroglucinol equivalents/g extract (mg PE/g); ^b^ radical scavenging activity (EC_50_ µg/mL); ^c^ µM of FeSO_4_ equivalents/g extract (µM FeSO_4_/g); ^d^ µmol of Trolox equivalents/g extract (µmol TE/g). EC_50_ values were determined for a 95% confidence interval. BHT (3,5-di-*tert*-4-butylhydroxytoluene).

**Table 2:** Enzymatic inhibitory activity (IC_50,_ µg/mL) of *Carpomitra costata* fractions and of the reference compounds, epigallocatechin gallate (ECGC) and kojic acid.

Fraction	Collagenase	Elastase	Hyaluronidase	Tyrosinase
F1	104.0 (93.5–115.6)	83.9 (73.4–95.9)	47.4 (45.2–51.3)	>200
F2	>200	>200	46.2 (44.1–49.5)	>200
F3	7.2 (6.6–7.7)	4.8 (4.5–5.2)	>200	85.9 (80.9–91.1)
F4	>200	174.8 (151.5–201.8)	>200	>200
F5	>200	>200	48.1 (45.6–51.0)	>200
EGCG	4.8 (4.1–5.5)	113.9 (80.7–160.0)	119.1 (126.1–320.4)	-
Kojic Acid	-	-	-	18.3 (14.0–23.9)

**Table 3:** Antimicrobial activity (IC_50_, µg/mL) of *Carpomitra costata* fractions and reference drugs (oxytetracycline and amphotericin B).

Fraction	*Staphylococcus epidermidis*	*Cutibacterium acnes*	*Malassezia furfur*
F1	>200	141.4 (119.8–169.1)	>200
F2	>200	45.9 (30.2–65.7)	>200
F3	>200	>200	>200
F4	>200	>200	>200
F5	72.0 (64.7–80.1)	46.3 (38.8–53.7)	>200
Oxytetracycline	12.4 (11.2–16.1)	0.07 (0.05–0.09)	-
Amphotericin B	-	-	11.4 (8.6–15.0)

## Data Availability

The data presented in this study are available on request from the corresponding author.
